# Systematic review of digital twin technology and applications

**DOI:** 10.1186/s42492-023-00137-4

**Published:** 2023-05-30

**Authors:** Jun-Feng Yao, Yong Yang, Xue-Cheng Wang, Xiao-Peng Zhang

**Affiliations:** 1 Center for Digital Media Computing, School of Film, Xiamen University, Xiamen 361005, China; 2 School of Informatics, Xiamen University, Xiamen 361005, China; 3 Key Laboratory of Digital Protection and Intelligent Processing of Intangible Cultural Heritage of Fujian and Taiwan, Ministry of Culture and Tourism, Xiamen 361005, China; 4 State Key Laboratory of Multimodal Artificial Intelligence Systems, the Institute of Automation, Chinese Academy of Sciences, Beijing 101408, China

**Keywords:** Digital twin, Digitalization, Virtual modeling, Information technology, Simulation

## Abstract

As one of the most important applications of digitalization, intelligence, and service, the digital twin (DT) breaks through the constraints of time, space, cost, and security on physical entities, expands and optimizes the relevant functions of physical entities, and enhances their application value. This phenomenon has been widely studied in academia and industry. In this study, the concept and definition of DT, as utilized by scholars and researchers in various fields of industry, are summarized. The internal association between DT and related technologies is explained. The four stages of DT development history are identified. The fundamentals of the technology, evaluation indexes, and model frameworks are reviewed. Subsequently, a conceptual ternary model of DT based on time, space, and logic is proposed. The technology and application status of typical DT systems are described. Finally, the current technical challenges of DT technology are analyzed, and directions for future development are discussed.

## Introduction

Digital twin (DT) technology is also known as digital avatars [1], digital masters [2], digital shadows [3], etc. It is a technology that accomplishes the mapping of the real world to the digital world and realizes the interaction between them in real time. This technology overcomes the constraints of real environmental factors. It can extend the relevant functions of the real world to the digital world and react to the real world. Currently, DTs are characterized by three functions [4]: (1) data fusion of various features of physical objects and high-fidelity real-time mapping of physical objects; (2) coexistence and coevolution throughout the lifecycle of physical objects; and (3) description, optimization, and control of physical objects.

**Fig. 1:**
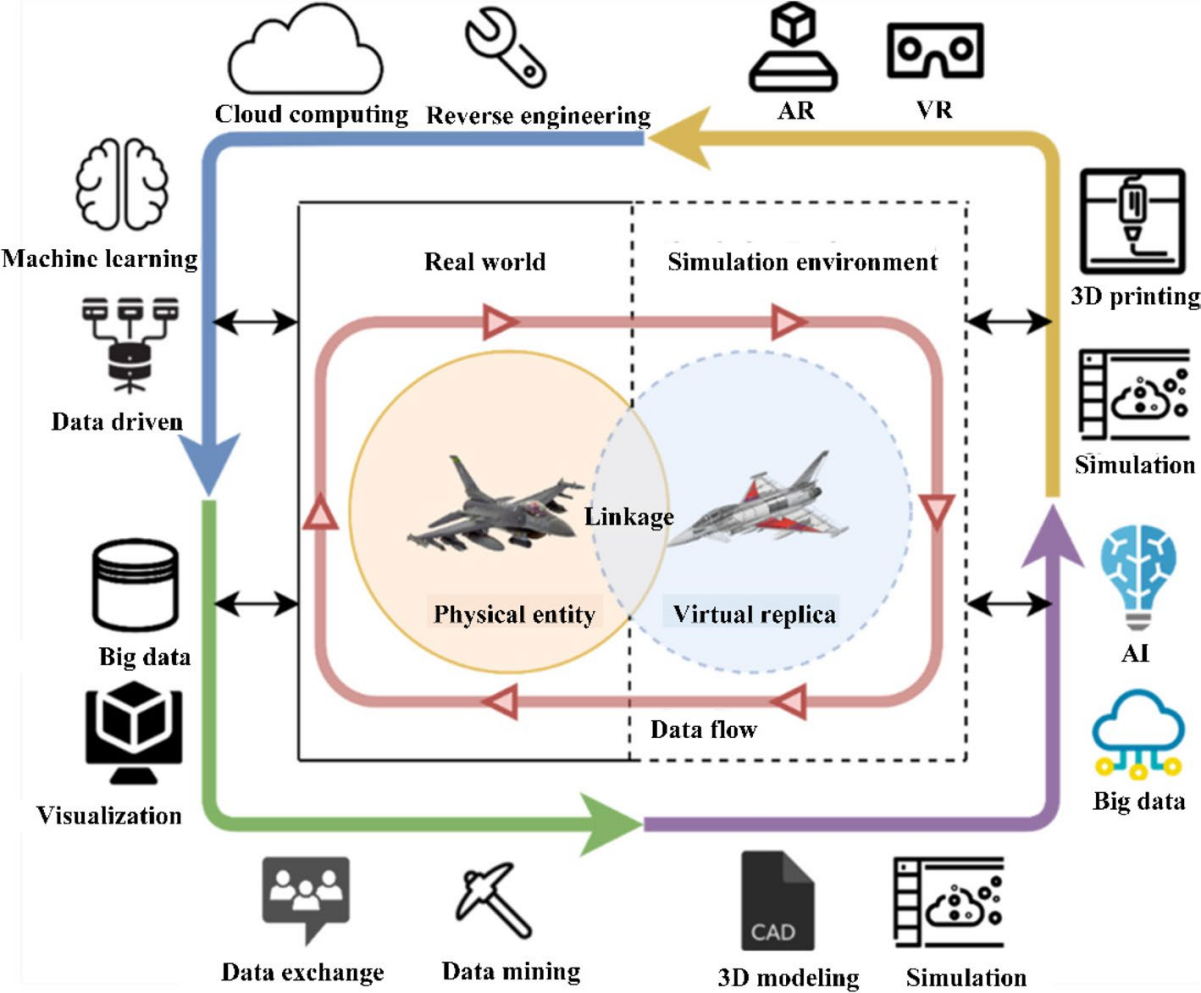
DT and related technologies [5]

DTs originated in the United States military aerospace industry. They have now been extended to transportation, industrial production, intelligent education, and other industry sectors. They can contribute to simulation, monitoring, evaluation, prediction, optimization, control, and other applications. As shown in Fig. 1, DT technology is strongly correlated with multiple technologies. DT is regarded as a key technology for realizing the digital transformation of enterprises and is also a hot technology of interest in industry and academia.

Although the concept of DT has been around for many years, it has not attracted much attention until the last few years. In 2019, academic exchange presentations at the “3rd Academic Conference on Digital Twin and Intelligent Manufacturing Services” further promoted the thinking of scholars in various fields on theories and technologies related to DTs [6]. In 2021, the first International Conference on Digital Twin Technology and first International Workshop on Model-driven Engineering with Digital Twin were held.

This study analyzes and reviews the research on DT technology and its applications. In “The concept of DT” section, the definition of DT in various domains is presented. The relationships and differences between DT and other related technologies are explained. In “The evolution of DT” section, the four stages and main processes of the DT development are clarified. In “The technical system of DT” section, the technical basis, evaluation indices, model framework and applications of the DT are introduced, whereby time, space, and logic model (TSL Model) of DT is proposed. Finally, the challenges and future trends are analyzed from the perspectives of DT technology and application research.

## The concept of DT

DT is an emerging technology concept, which foremost has practical utility succeeded by concept. DT exhibits the typical characteristics of cross-technology fields, cross-system integration, and cross-industry integration. The technical scope of this study is wide. There is a strong correlation and continuity between computer-aided technology, simulation systems, extended reality (XR), metaverse, and other technologies.

### Definition of DT

The concept of a DT was introduced by Grieves and Vickers [7] in 2013, and since then, the definition of DT has proliferated, as listed in Table 1. However, the wide range of DT systems preclude the development of a unified definition for DTs [8]. Researchers have mainly defined DT from one or more perspectives, such as models, data, links, and functions.

**Table 1:** The definition of DT

No.	Reference	Year	DT definition
1	[9]	2012	DT is a multi-scale integrated simulation of a physical equipment or system that makes full use of virtual models, real-time sensor data, and historical data on the equipment or system to map the entire life cycle of the process
2	[8]	2012	DT models need to meet the requirements of the system and subsystems throughout their life cycle, enabling the assessment of the vehicle’s mission accomplishment capabilities
3	[10]	2013	DT is an approach that uses data-driven analytical algorithms and other physical models to simulate the operational state of an entity, which can be described as a 5S system consisting of sensing, storing, synchronizing, synthesizing, and serving
4	[11]	2015	DT comprises a large collection of digital products, requiring a good architecture to link all components
5	[7]	2017	DT requires not only virtual mapping of products at the macro-geometric level, but also all the information about the actual manufactured products at the micro-atomic level
6	[12]	2017	DT of a product needs to realize the full-factor digital mapping of physical entities in virtual space, to simulate, predict, and control the feedback of physical entities
7	[13]	2018	DT is a real time mapping of physical, virtual, and interaction data in the entire life cycle of a physical entity
8	[14]	2018	DT is an all-encompassing digital representation of a product that simulates the properties, conditions, and behavior of an actual physical object through data and models
9	[15]	2019	DT is a dynamic virtual model that digitally builds multi-dimensional, multi-physical entities of multi-temporal scale to reflect the properties and behaviors of the real environment
10	[16]	2019	DT is a simulation process that integrates multiple physical quantities and scales in the entire life cycle of physical equipment, using physical models, real-time sensor data, operational history data, etc., to build models in virtual space to complete the mapping
11	[17]	2022	The twin is a refined digital description of the product entity. Simulation experiments based on digital models can more realistically reflect the characteristics, behavior, formation process, and performance of physical products. The real-time collected data are associated and mapped onto the DT to identify, track, and monitor the product. The DT can predict and analyze the behavior of simulated objects, diagnose faults and issue warnings, locate and record problems, and achieve optimal control

DT was defined from a model-focused perspective in 2012 by NASA as a multi-scale integrated simulation of a physical equipment or physical system that makes full use of virtual models, real-time sensor data, and historical data to map the entire life cycle process of the equipment or system [9, 18]. Ríos et al. [19] introduced this concept into the product design process and extended DT to the general industrial field. Han [20] summarized the related literature to define DT as a digital model for describing the full lifecycle information of physical entities, including the precise mapping relationship between digital voxels and physical entities. Grieves and Vickers [7] argued that a DT requires not only virtual mapping of products at the macro-geometric level but also all the information about actual manufactured products at the micro-atomic level.

DT was defined from a data-focused perspective in 2013 by Lee et al. [10] as an approach that uses data-driven analytical algorithms and other physical models to simulate the operational state of an entity. This can be described as a 5S system consisting of sensing, storing, synchronizing, synthesizing, and serving.

DT was defined from a function-focused perspective by Zhuang et al. [12] who added the main functions of a DT to the original definition, emphasizing that the DT of a product must realize full-factor digital mapping of physical entities in virtual space, to simulate, predict, and control the feedback of physical entities. Nie et al. [17] defined DT as a refined digital description of a product entity. Simulation experiments based on digital models can more realistically reflect the characteristics, behavior, formation process, and performance of physical products. A DT can interact with reality, by associating and mapping real-time collected data to identify, track, and monitor products. Simultaneously, the DT can predict and analyze the behavior of simulated objects, diagnose faults and issue warnings, locate and record problems, and achieve optimal control.

DT was defined from a linkage-focused perspective by Rosen et al. [11] who argued that a DT not only comprises a large collection of digital products but also should possess a good architecture so that all components are linked.

To summarize the concept and understanding of the DT, DT is defined as a digital mapping of the physical world in TSL. This reflects the composition and structure of entities, relationship between entities and the external environment, and developmental process of entities in the digital world, thereby enabling the acquisition of the current state of entities, prediction of subsequent changes in entities, and guidance in the operation of entities.

### DT technology connection

DT technology is closely related to computer-aided technology, virtual simulations, XR, and metaverse technology, which have a lot in common but also differences in technical focus.

#### The association of DT with computer-aided technology

Computer-aided technologies include computer-aided design (CAD) [15, 16, 21], computer-aided engineering (CAE) [22], and other methods which use computers and graphic devices to assist designers with rapid retrieval, editing and processing, solution comparison, and other related tasks.

Computer-aided technology has many of the same technical requirements as DT technology; however, there are also significant differences. Both CAD/CAE and DT models are required to achieve high fidelity, reliability, and accuracy. CAD/CAE models can be two- or three-dimensional, whereas DT models must be three-dimensional. CAD/CAE models are usually static and limited to a certain process of the project, whereas DT models are used throughout the system life cycle. CAD/CAE models are used to verify product performance, simulate manufacturing processes, and validate design feasibility without interaction, whereas DT models are interactive in terms of data feedback and control and are useful for enhancing the traditional product design and development process.

#### Association of DT with virtual simulation technology

Virtual simulation technology [23–26] is a computer system for creating and experiencing a virtual world. A virtual simulation can be either a replication of the real world or a separate concept of the real world.

Virtual simulation is one of the core technologies of DT but is fundamentally different from DT. Simulation technologies partially reproduce the real world offline, primarily during the research and design stages. They usually do not perform analyses or optimization functions. However, DT reflects the state changes of physical objects in real time and can be used to analyze and predict the decision optimization function of physical entities. Simulation technology relies on models and data to map the properties and parameters of the physical world. DT must sense, diagnose, and predict the state of physical entities in real time to optimize them.

#### Association of DT with XR

XR [27, 28] is a human-computer interaction-based virtual reality (VR) environment generated by computer technology and wearable devices. XR is a generic term for VR [29, 30], augmented reality (AR) [31, 32], and mixed reality [33]. VR is the simulation of a completely virtual digital world using devices, such as VR eyes and gamepads, which provide users with both visual and auditory sensory experiences. AR is an integrated technology that combines real-world and virtual-scenes. By implanting specific images or information from the real world into a program, and by upgrading, supplementing, and rendering the content, the information processed by the computer is used to simulate a specific scene and overlaid onto the real-world image.

XR improves user experience through the integration of virtual and real environments, whereas DT is accompanied by the entire life cycle of the process, thereby focusing on the development and change of entities, to close the loop with reality.

#### Association of DT with metaverse

The concept of metaverse [34] is not yet universally defined although interest in metaverse exploded in 2021, which is generally considered to be the first year of metaverse research. Metaverse is an emerging cross-cutting field involving philosophy, economics, management, education, and computer science, among others [35–37].

The participants in the metaverse group are real people with dynamics, highly civilized, and socially interactive. The objects of the metaverse are digital simulations of real objects as well as special objects with no real counterpart. A DT is a simulation of the entire life cycle of development, focusing on virtual-real interactions, where each digital object has a corresponding objective counterpart.

Therefore, DT technology is closely related to the above four closely related areas that complement each other.

## The evolution of DT

**Fig. 2:**
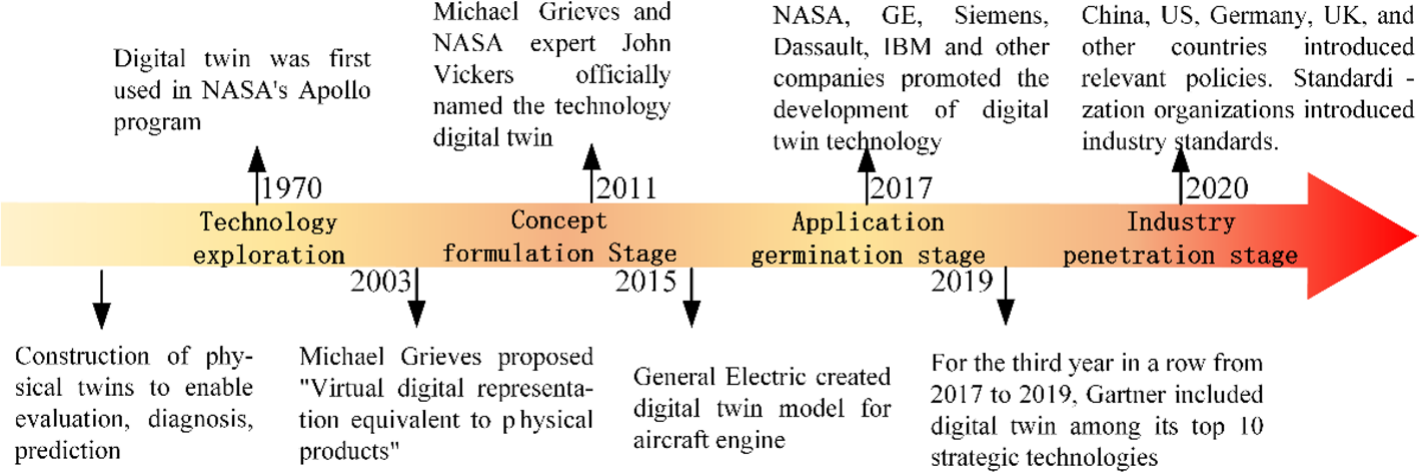
The evolution of DT

DT originated in the aerospace industry. With the development of new generation information technology, DT has experienced four developmental stages: technology exploration, concept formulation, application germination, and industry penetration (Fig. 2).

### Technology exploration stage

The initial development of twin technology involved building twins of physical entities to evaluate, diagnose, and predict physical entities. In 1970, after NASA launched Apollo 13, the spacecraft experienced severe malfunction. For a successful rescue operation, physical replication systems were created on the ground to match the spacecraft, and to train astronauts and mission controllers for each mission scenario [38]. However, this approach has three main shortcomings: (1) Physical entities are strictly unique. Therefore, complete consistency between physical entities and physical twins cannot be guaranteed; (2) Creating twins of physical entities is costly, incurring high trial and error cost for physical twins; and (3) The real-time interaction between physical entities and twins is poor. Therefore, a rapid response to state changes of physical entities cannot be achieved.

With the development of computers and related technologies, researchers attempted to build digital virtual entities to improve the performance of physical entities through feedback. In 1970, NASA built a semi-DT system for the Apollo program to train personnel and troubleshoot for space exploration. During the training process, the mission console and cockpit were physical entities replicated from the spacecraft, whereas the command module, lunar module, and other equipment were digital virtual objects created by multiple computer simulations. Although the system could not be fully digitized because of the limitations of technology at that time, this is still considered the earliest application of DTs [39] despite the fact that DT technology was not widely used at the time.

### Concept formulation stage

The concept of DT was first introduced by Professor Grieves at the University of Michigan. His concept of DT and the corresponding model were important in leading the development of this technology.

In 2002, Professor Grieves proposed the creation of physical products, virtual products, and a data interface between them. This is a vision of DT in the context of product life cycle management. In 2003, Professor Grieves proposed the concept of a virtual digital representation equivalent to the physical product [7]. In the period from 2003 to 2005, Professor Grieves referred to this vision as “Mirrored Spaced Model” [40]. From 2006 to 2010, “Information Mirroring Model” [41] was used to describe this vision; however, it was not until 2011 that Professor Grieves and NASA expert John Vickers co-named digital twin. The two researchers proposed a DT 3D model that combined real space, virtual space, and the data flow connection between them, considering the actual situation at the time [42]. Although Professor Grieves actively explored DTs and related technologies, few researchers focused on DT-related technologies because of the limitations of the internet of things (IoT) and data processing technologies at that time, limiting wide implementation and use.

### Application germination stage

**Fig. 3:**
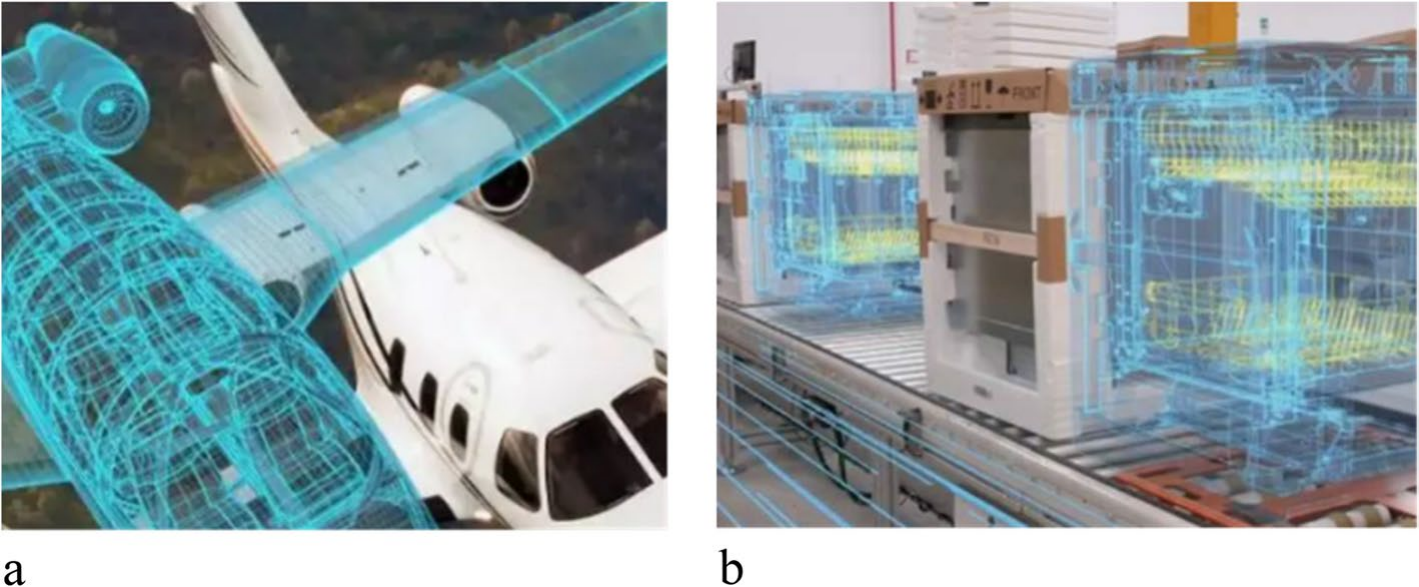
DT applications. **a** Digital companion flight; **b** Digital factory

DT technology applications have emerged in industry since 2010. As shown in Fig. 3, typical industrial applications are primarily in system operations, entity management, and manufacturing.

The first application of DT was in operational control of aviation systems. In 2010, the United States military implemented digital companion flights for F35 based on DT technology, to reduce aircraft maintenance and utilization costs. In 2011, the United States Air Force Research Laboratory introduced DT technology for aircraft health control and achieved significant results [9, 43]. In 2015, General Electric built a twin model for passenger aircraft engines to enable real-time monitoring and predictive maintenance.

The second application of DT involved the physical management of large installations. In 2017, General Electric used DT technology to achieve virtual inspection and simulation of equipment and processes for better management of entities, such as power plants and turbine engines. In the same year, Siemens AG integrated DTs into management of assets, product lifecycle, and manufacturing processes on top of the industrial Internet to achieve closed-loop optimization and scheduling of multiple DT systems.

The third application of DT was for the interaction design of complex device manufacturing. In 2017, Dassault used DT technology to enable product interaction design, testing, and optimization, allowing designers and customers to predict the effects of products before they are created, thereby improving the products based on digital objects [44, 45].

DT technology is rapidly developing in industrial manufacturing, driven by NASA and companies such as General Electric, Siemens AG, and Dassault.

### Industry penetration stage

**Fig. 4:**
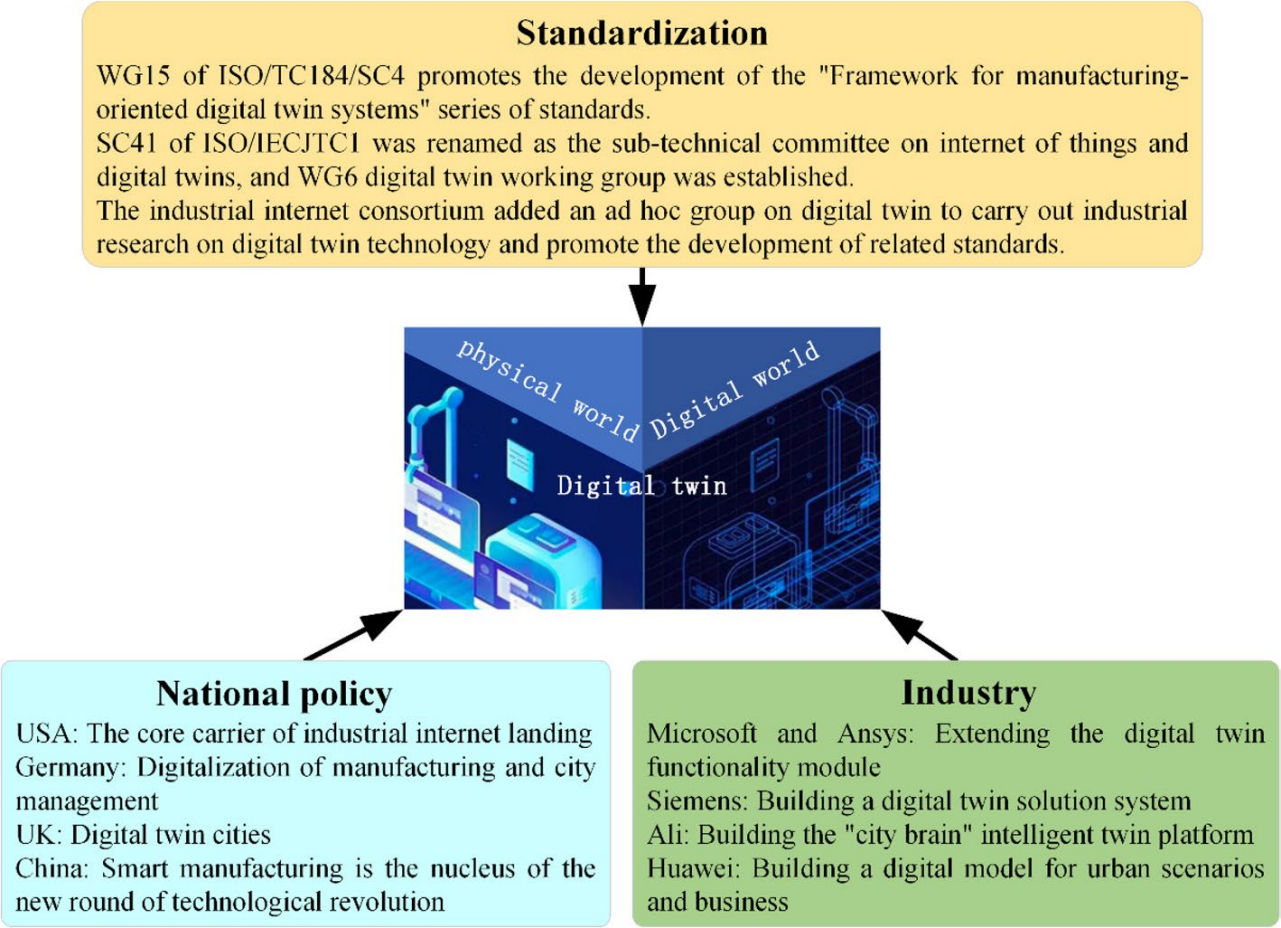
Policy support and industry penetration of DT

With the further development of computer and network technologies, the application of DT has been gradually extended to various industries with published research results. Gartner Consulting listed it as one of the top 10 strategic technologies for 2017–2019. Concurrently, national policies, industrial applications, and standardization related to DTs emerged (Fig. 4).

In terms of national policies, the United States has adopted DT as the core carrier for the implementation of the industrial Internet, focusing on applications in the military and large equipment sectors. Germany promotes asset management shells under Industry 4.0 architecture, focusing on the digitalization of manufacturing and city management. The United Kingdom has established the Digital Construction UK Centre, targeting DT cities and creating national twins. In 2020, the United States Industrial Internet Consortium and Germany’s Industry 4.0 platform jointly released a white paper on DTs, incorporating DTs into the industrial IoT technology system. Since 2019, the Chinese government has issued several relevant documents to promote the development of DT technology. The 14th Five-Year Plan explicitly states that DT technology must be developed to realize the construction of digital China. DT technology has been listed as one of the top ten technological advances in smart manufacturing [46].

In industry, Microsoft partnered with Ansys to extend the DT function module to the Azure IoT platform. Siemens built a complete DT solution system based on an industrial Internet platform that incorporated mainstream products and systems. Ansys relies on DT technology to model the entire life cycle of complex product objects, along with simulation analysis to open up data flow from product design and development to production. Alibaba aggregated multi-dimensional data from cities to build a “city brain” intelligent twin platform, providing an integrated solution for smart parks, which was implemented in the Xiaoshan District, Hangzhou. HUAWEI released the Wotu digital twin platform to create a digital innovation model for urban scenarios and businesses empowered by 5G + AI.

In terms of standardization*,* to promote the construction of DT standards and initiate proof-of-concept projects, international standardization organizations such as ISO, IEEE, IEC, and ITU have established technical committees and working groups. To better promote the international standardization of DTs, WG15 of ISO/TC184/SC4 developed and validated a framework series of standards for manufacturing-oriented DT systems. In 2020, ISO/IECJTC1 established the WG6 Digital Twin Working Group. At about the same time, the Alliance for Industrial Internet established an ad hoc group on DTs.

## The technical system of DT

Based on an analysis of the development history and concepts of DTs, it was determined that DT displays four typical technical features: virtual-real mapping, real-time synchronization, symbiotic evolution, and closed-loop optimization. Researchers have actively explored various fields to implement DT systems for different types of tasks.

### DT technology fundamentals

#### Data acquisition and transmission technology

A DT is a real-time dynamic surreal mapping of a physical entity system. Real-time data acquisition, transmission, and updating, play crucial roles in DTs. Numerous distributed high-precision sensors of various types are at the forefront of the entire twin system and play a fundamental sensory role in the entire twin system. The distribution of sensors and the construction of sensor networks are based on the principles of fast speed, safety, and accuracy, whereby distributed sensors are used to collect various types of physical quantity information on the system to characterize the system state [47].

The more accurate the data returned by the sensors, the better is the simulation of the DT system resulting in more accurate simulation states and effects. DT interaction is a multi-dimensional and multi-timescale coupling process with the ultimate goal of controlling reality through the virtual environment. However, differences in coding formats between multiple-source sensors, make it difficult to avoid data errors in the process of mutual fusion.

Presently, the specific difficulty of data acquisition in DT systems is that the type of sensor, accuracy, reliability, and working environment are limited by the current level of technological development, consequently limiting data collection methods. The key factors in data transmission are real-time speed and security. However, network transmission equipment and network structures are limited by the current level of technology, which cannot meet the higher level of transmission rates. Attention should also be paid to network security in practical applications.

#### Lifecycle data management

The entire data storage and management of complex systems provide an important support to DT systems. Cloud servers for the distributed management of massive system operational data enable high-speed data acceptance and secure redundant backup, providing sufficient and reliable data sources for intelligent data analysis algorithms, which play an important role in maintaining the operation of the entire DT system [48]. By storing the entire lifecycle data of the system, sufficient information can be provided for data analysis and presentation, enabling the system to perform the functions of historical state playback, structural health degradation analysis, and intelligent analysis of any historical moment. The large amount of historical data also provide rich sample information for data mining, which can be used to obtain many unknown but potentially valuable pieces of information on the data analysis results, with a deeper understanding and cognition of system mechanisms and data characteristics, to realize the surreal properties of DTs.

The implementation of full lifecycle data storage and management require distributed storage with the help of servers. Because DT systems require large amounts of real-time data, optimizing the data distribution architecture should be the main task, to ensure real-time and reliable data reading performance of storage and retrieval methods, which is a challenge in DT system applications. Considering the data security of industries and information protection in terms of equipment, building a data center or data management system with a secure private cloud as the core is currently a more feasible technical solution.

#### High-performance computing

The implementation of the complex functions of a DT system relies heavily on the computing platform. Real-time performance is an important indicator of the performance of DT systems. Optimizing the data and algorithm structures to improve the task execution speed of the system is important in ensuring the real-time performance of the system. In DT applications, it is important to consider the comprehensive performance of the system computing platform, the time delay of the data transmission network, the computing capacity of the cloud computing platform, and the design of an optimal system computing architecture that satisfy the real-time analysis and computing requirements of the system. The level of the digital computing capability of the platform directly determines the overall performance of the system and undoubtedly the computing foundation of the entire system.

#### Virtual modeling and simulation technology

High-fidelity virtual modeling technology is the ‘soul’ of DTs. A dynamic simulation reflects the fact that DT is a dynamic process that spans the entire product lifecycle. The high-fidelity virtual modeling and dynamic simulations of a DT are designed to restore the various geometric rules and physical properties of relative entities in the computer as much as possible.

For high-fidelity virtual modeling and dynamic simulation, first, the fusion of multi-domain, multi-dimensional, multi-timescale, and high-precision model data are required. Second, the system must be able to monitor the simulation process in real time and obtain feedback data for complete self-update and optimization. Multidomain modeling is another important aspect that refers to the cross-domain fusion modeling of physical systems from different domain perspectives under normal and abnormal operating conditions. Multidomain modeling implementation starts with the initial conceptual design stage to understand and model the fusion design at a deep mechanistic level [49].

Most current modeling approaches involve model development and maturation in specific domains. Integration and data fusion methods are then used at a later stage to fuse the independent models from different domains into a comprehensive system-level model. However, this fusion approach does not have sufficient depth of integration and lacks reasonable explanation, limiting the ability to deeply fuse models from different domains. The difficulty of multi-domain fusion modeling is that the fusion of multiple characteristics leads to a large degree of freedom in the system equations, whereas the data collected by sensors require a high degree of consistency with actual system data to ensure dynamic updating of the model based on high-precision sensing measurements.

#### Other key technologies

A DT system is characterized by numerous parameters, large data redundancy, and unavoidable and complicated types of noise. These parameters are characterized by strong coupling, nonlinearity, and time variation that directly affect the quality of the data, which is the key for building DT models. Therefore, there is an urgent need to develop efficient big-data processing techniques.

Visualization techniques for DT systems are regarded as the most effective means of understanding useful information for making decisions, which are of considerable importance in building a DT system. It is difficult for traditional visualization methods to directly deal with the explosive growth of big data to express the meaning and value hidden in the data in a timely and effective manner.

Artificial intelligence techniques drive the development of DT technologies. Considering the essential differences between commercial and industrial big data, intelligent aspects, such as abnormal or fault state simulation and injection should be considered in the quantitative analysis of industrial data for enhanced deep learning with little or no samples. All of these aspects are current research features or challenges in data generation, data analysis, and modeling.

### DT evaluation index

With the development of DT technology, DT models have become more diverse, placing greater demand on the transparency of DT model performance. The main problem, however, is the lack of systematic evaluation theories and methods to guide the construction and verification, operation and management, reconfiguration and optimization, migration and reuse, as well as circulation and delivery of DT models. This problem makes it difficult to analyze and quantify the quality, performance, applicability, symbiosis, adaptability, and value of DT models, seriously hindering the in-depth promotion and application of DTs.

**Fig. 5:**
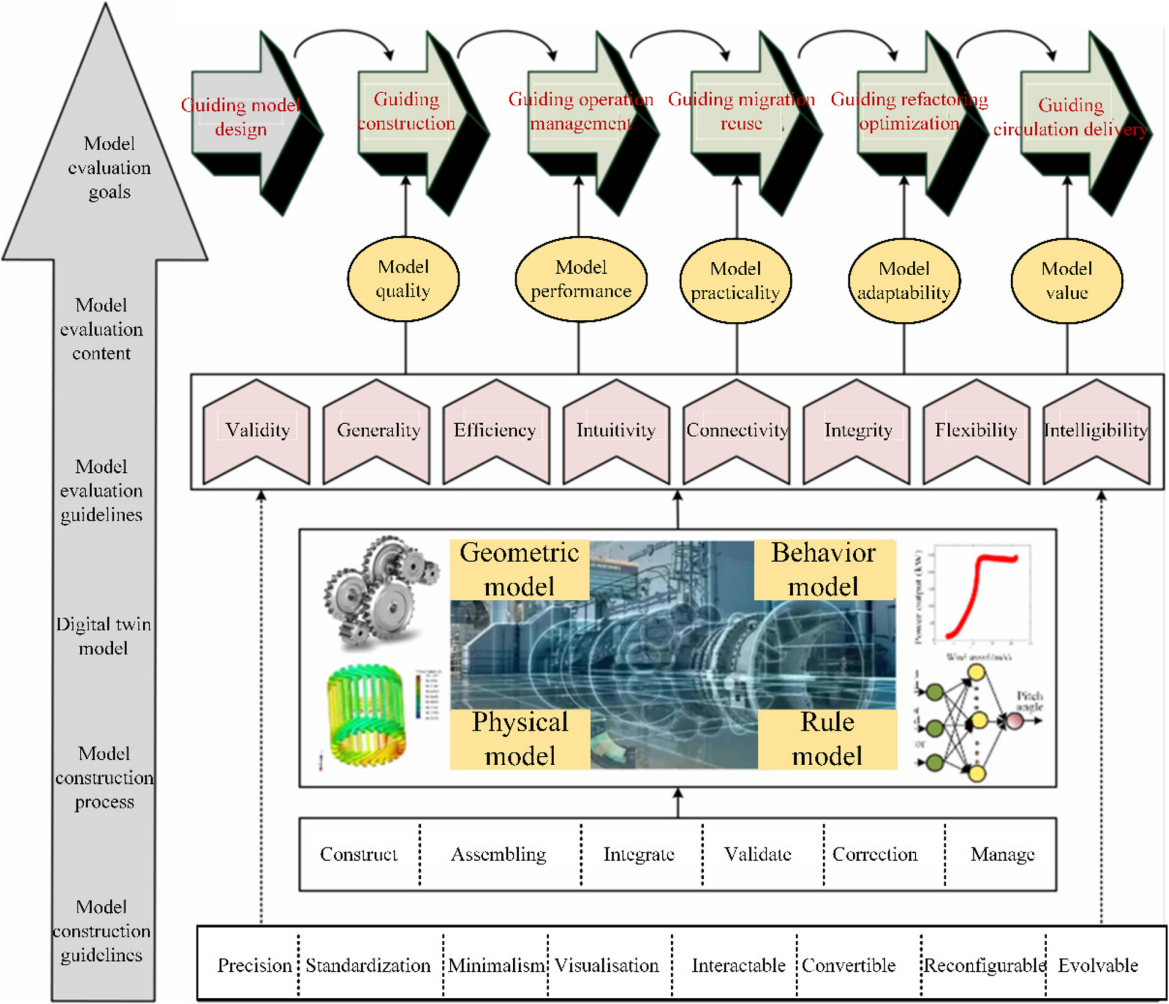
DT model evaluation index system [50]

Zhang and Tao [50] following the principles of scientificity, generality, comparability, and operability, proposed a quantifiable and targeted construction of an evaluation index system. The effectiveness, generality, efficiency, intuitiveness, connectivity, wholeness, flexibility, and intelligence of the DT model were established as evaluation criteria. The evaluation index system consisted of one total index, eight secondary indices, and 29 tertiary indices, as shown in Fig. 5.

**Fig. 6:**
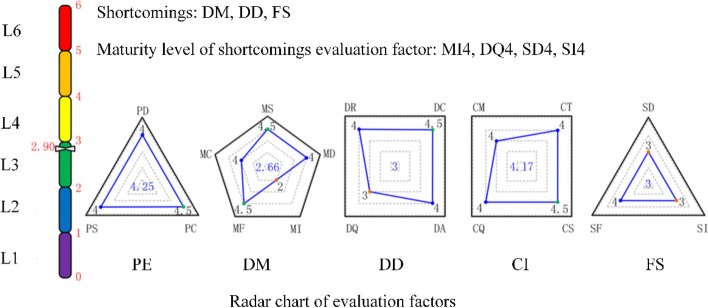
Maturity evaluation of DT robots [51]

Tao et al. [51] statistically analyzed existing theoretical research and application practices related to DTs. DTs are classified into the following six categories on their functions and uses: (1) DT-based physical entity design verification and equivalence analysis, (2) DT-based physical entity operation process visualization and monitoring, (3) DT-based physical entity remote operation and maintenance control, (4) DT-based diagnosis and prediction, (5) DT-based intelligent decision-making and optimization, and (6) DT-based physical entity entire life cycle tracking, retracing, and management. By analyzing the commonalities of the aforementioned types of DT research and applications, DTs are classified into six maturity levels on the functional services they provide according to the different connection interaction methods and automation degrees (Fig. 6).

**Fig. 7:**
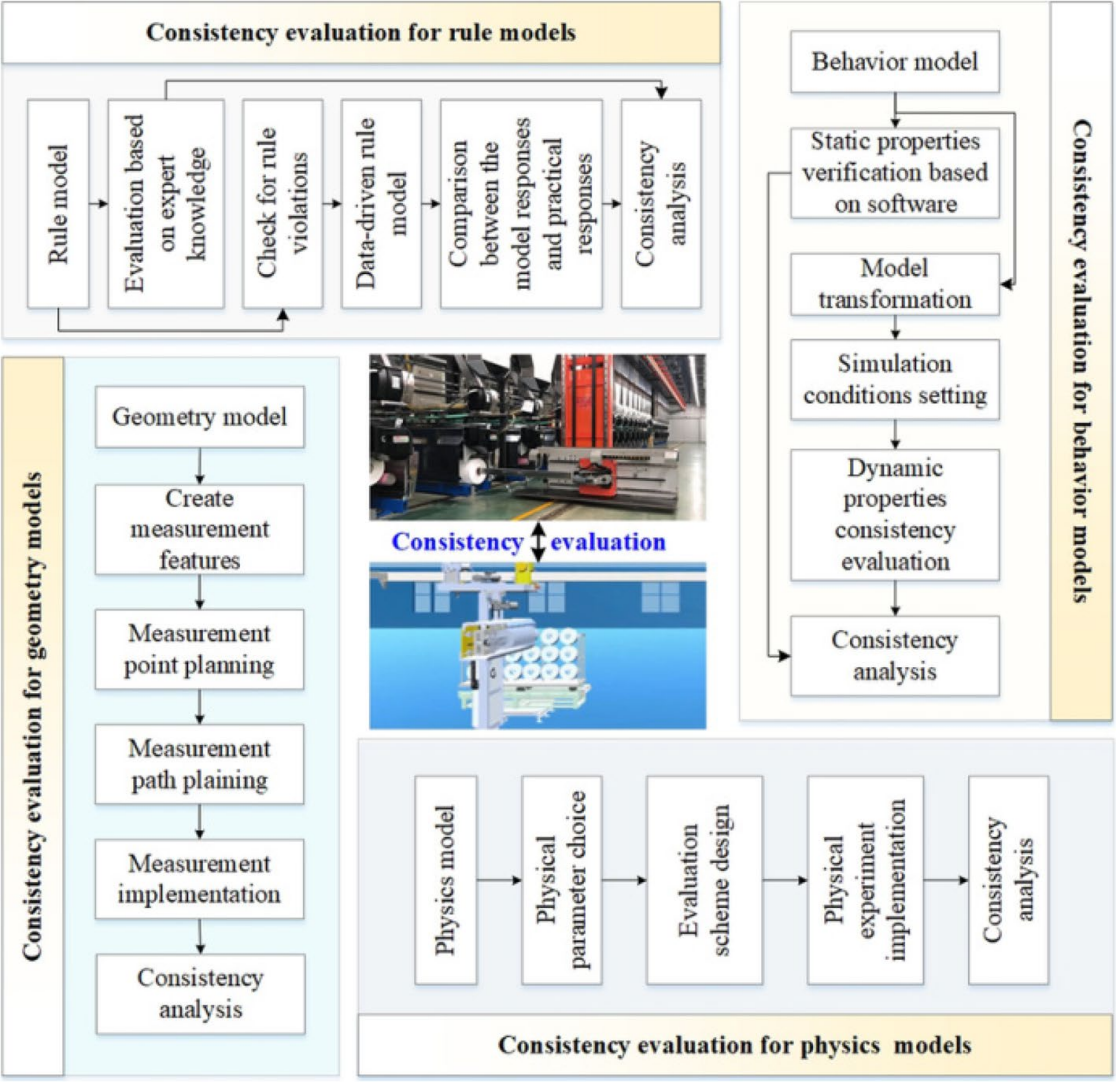
The consistency evaluation method for multi-dimension models

Zhang et al. [52] proposed a consistency evaluation method for DT shop models, considering the two main stages of before and after model assembly and model fusion. Before model assembly and fusion, consistency evaluation methods are discussed for geometric, physical, behavioral, and rule models. After model assembly and fusion, the study indicates how to determine whether the introduced relations are correct and accurate. A comprehensive evaluation of DT shop model is performed using hierarchical analysis (Fig. 7).

### DT model framework

DT framework research began in 2015. Kraft [53] proposed a DT analysis framework for the United States Air Force, providing engineering analysis capabilities and decision making support throughout the lifecycle of an air vehicle. A DT merges physical modeling and experimental data to generate an authoritative digital representation of the system at each stage of weapon system equipment and operation. Qi et al. [54] illustrated and highlighted a framework for a DT in manufacturing service by examining how manufacturers use various components of DTs in the form of services. Malik and Bilberg [55] proposed a framework to support a DT framework for human-machine co-design and build control. Xiao et al. [56] proposed and explored modeling concepts, methodological ideas, and theoretical frameworks based on DT systems for strategic enterprise scenarios based on smart manufacturing.

**Fig. 8:**
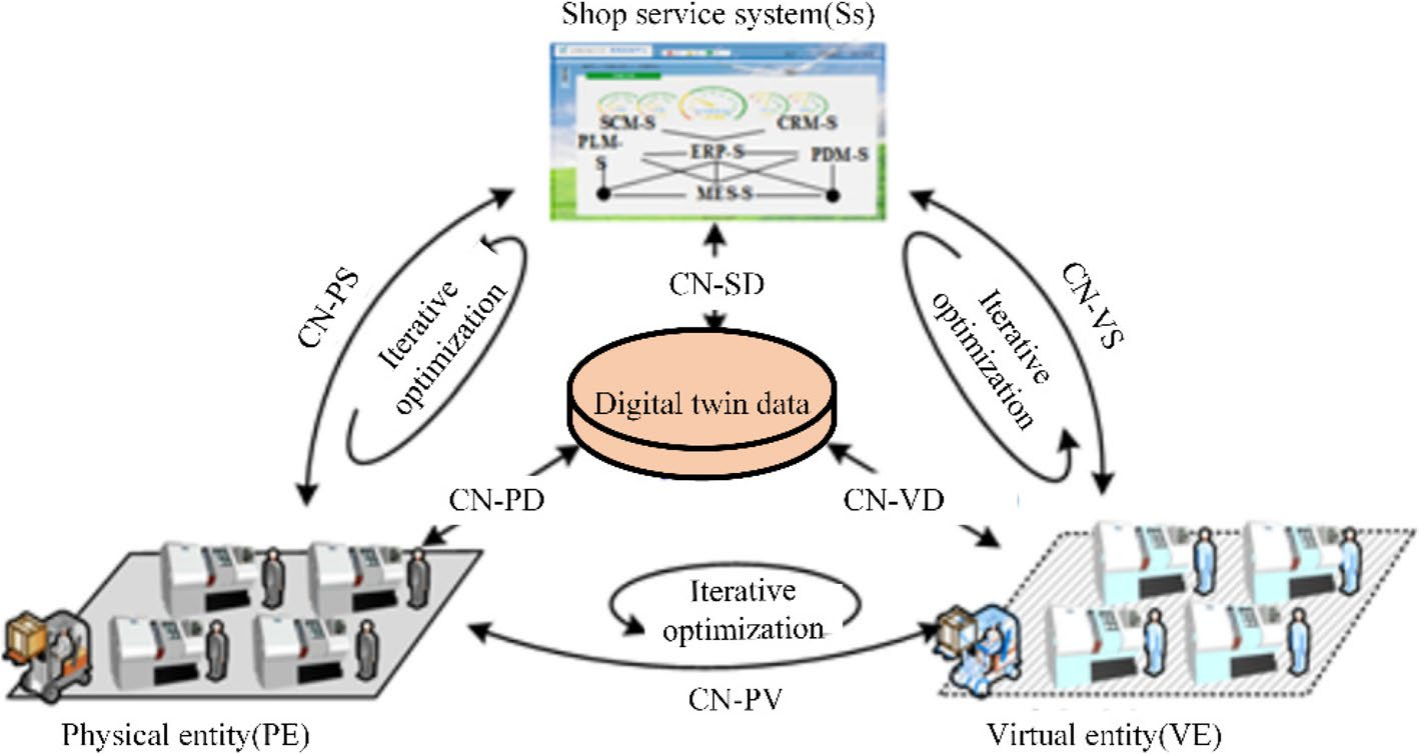
The five-dimensional DT model [42]

The Beijing University of Aeronautics and Astronautics digital twin research team created a five-dimensional DT model [13, 57–63] based on Professor Grieves’ three-dimensional model [42]. The five-dimensional DT model is expressed in Eq. 1:

1 $${M}_{DT}=\left(PE, VE, Ss, DD, CN\right)$$

where PE refers to the real physical entity; VE refers to the virtual equipment created using a computer; Ss refers to the services provided by the DT; DD refers to the data collected by various types of sensors; and CN refers to the connection between the components (Fig. 8).

**Fig. 9:**
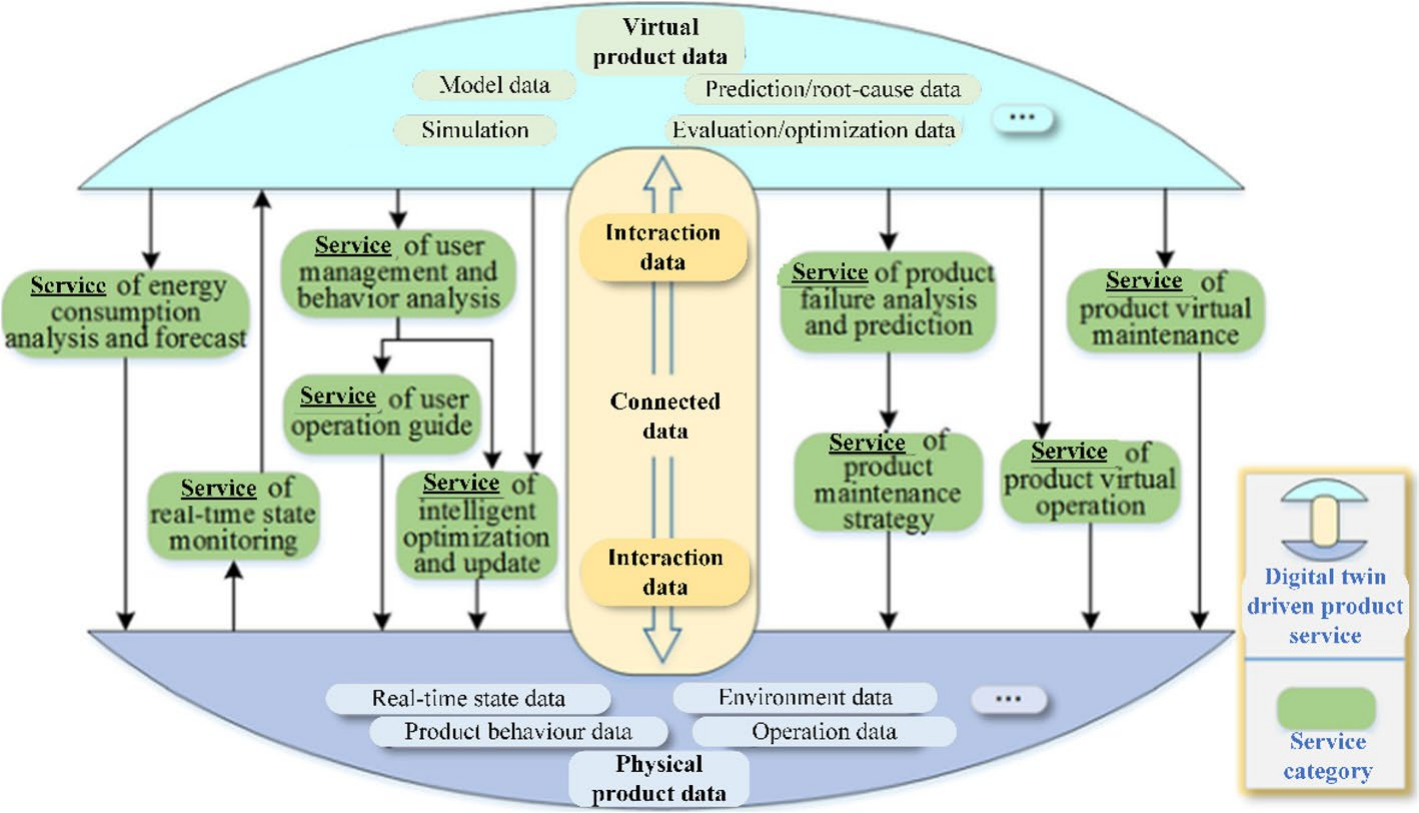
Services related to complex products [13]

The M_DT_ is a generic reference architecture for different applications in different domains. Xu [64] applied a five-dimensional model to the proposed edge-computing-based dynamic scheduling model (ECDTJ-DC model) of a DT workshop manufacturing process, along with a data collection and analysis model and dynamic scheduling knowledge model, both based on ECDTJ-DC for a DT workshop. Tao et al. [13] used a DT framework to understand degradation and anomalous events, to predict unknown events in advance. Thus, services related to complex products are provided to users and manufacturers, including services in the nine categories (Fig. 9).

**Fig. 10:**
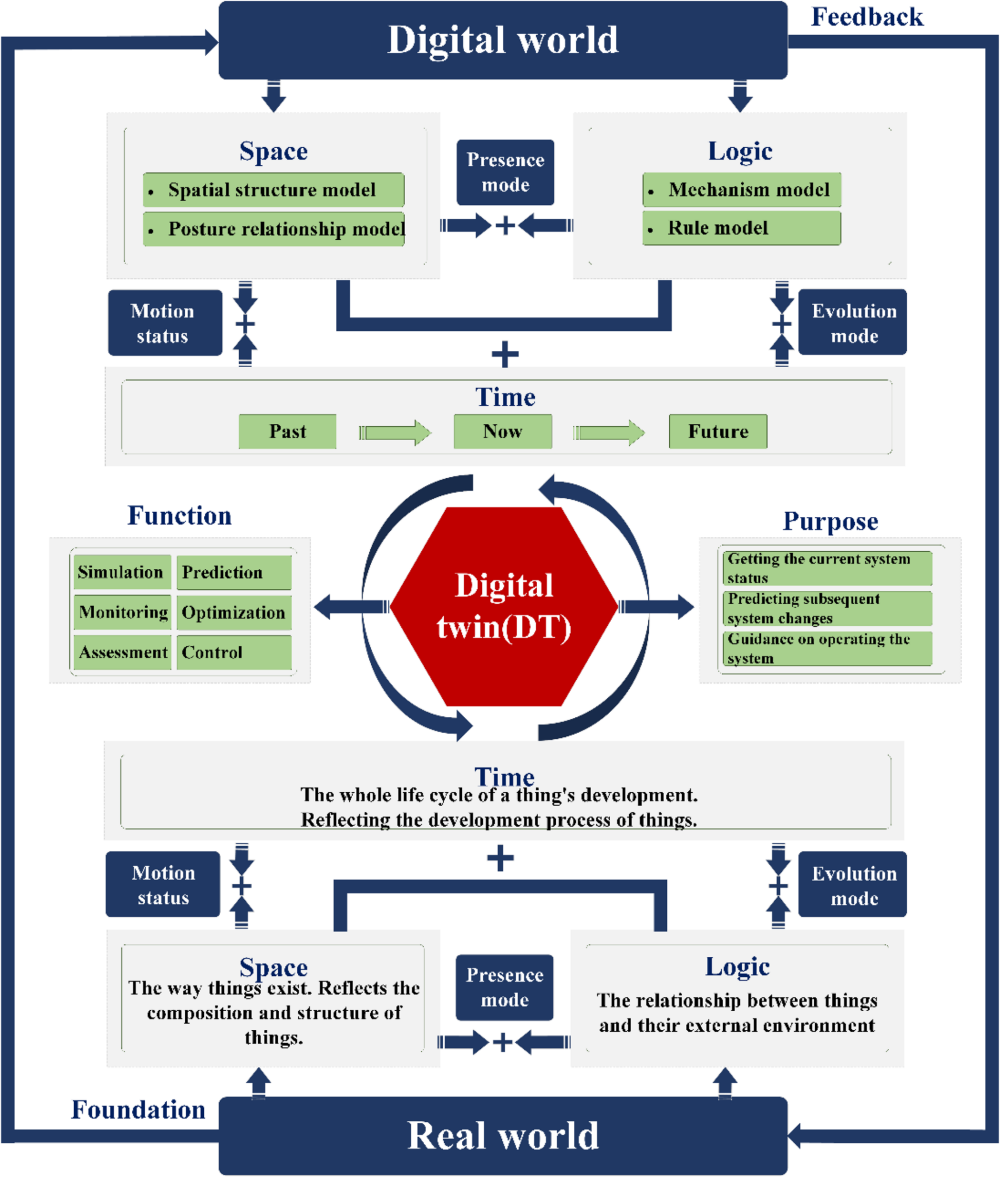
The TSL model of DT

The TSL ternary model of DT is proposed on the current analysis and practical requirements (Fig. 10). The model uses the concept of DT theoretical framework on TSL model.

2 $$DT=\left(T,S,L\right)$$

where T refers to the time element reflecting the entire lifecycle of the development of the object, including white, grey, and black data at different time points; S refers to the spatial element, reflecting the composition and structure of the object, including the geometric structure and its positional relationship model; L refers to the logical element reflecting the relationship between the object and the external environment, including the mechanism and rule models set by the user according to needs and experience; (T, S) refers to the combination of space and time, reflecting the movement of objects; (T, L) refers to the logic and time that reflects the evolution of objects; (S, L) refers to the space and logic reflecting the way objects exist.

The purpose of DT is to obtain the current state of the system, predict subsequent changes of the system, and guide its operation. Depending on the purpose of the DT, six functions can be derived: (1) Simulation: virtual testing, virtual verification, and operation preview; (2) Monitoring: operation monitoring, status monitoring, and fault diagnosis; (3) Evaluation: performance and status evaluation; (4) Prediction: quality, fault, performance, and life prediction; (5) Optimization: design, configuration, performance, and process optimization; and (6) Control: operation, remote, and cooperative control.

The five-dimensional DT model was derived in a DT workshop and gradually extended to other fields of application. This provides a common reference model for application support of DTs in different fields. However, this model lacks the classification and elaboration of model types and cannot reflect changes in models over time. The TSL ternary model is a virtual model derived from the spatial model of a real object, such as the geometric structure and positional relationship, and logical model, such as the mechanism and rules. The TSL ternary model emphasizes the time-varying property of the model to make it more consistent with the actual situation so that the physical world can be fully portrayed in the digital world.

#### Case: DT iron-making blast furnace

Blast furnace smelting is an important process in steel production. The interior of the blast furnace consists of four phases: gas, powder, liquid and solid. Coupled with complex heat, mass, and chemical reactions, the blast furnace is recognized as one of the most complex metallurgical reactors. The operation status of the blast furnace plays an important role in an enterprise’s safe production, cost reduction, and efficiency. In the field of blast furnace smelting, many challenges remain: (1) Establishing a high-precision DT of the complex interior of a blast furnace based on the ternary expression of mechanism, data-driven, and geometric models; (2) Establishing a library of industrial boiler DT instances for the large blast furnace structure with numerous sensors and a large amount of historical operation data; and (3) Visualization of blast furnace status monitoring and improvement in real-time interaction.

**Fig. 11:**
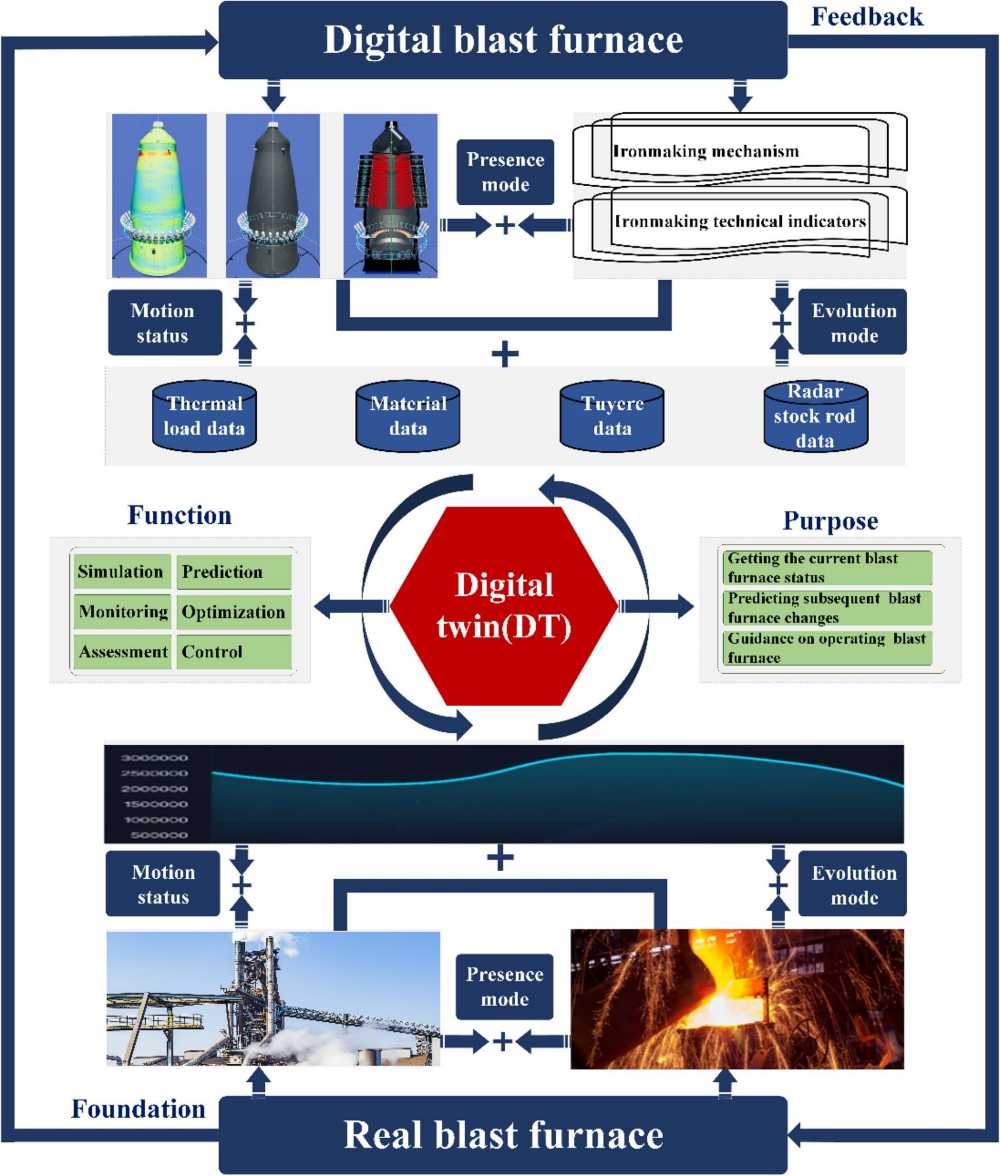
DT blast furnace based on TSL model

The authors applied a new generation of information technology to build a DT model of an iron-making blast furnace on a steel company project (Fig. 11). The space-model was modeled on an equal scale by scientific calculations in strict accordance with the design drawings and on-site field mapping. In the logic-model, heat transfer, mass transfer, chemical reaction, and rule models of coke, coal, and fuel ratios in the steel smelting process are established. In the time-model, white, grey, and black data at different time nodes are saved. The DT realizes several functions, such as simulation of steel smelting, operation monitoring of steel smelting, state evaluation of blast furnace, prediction of steel smelting quality, configuration optimization of smelting raw materials, and collaborative control in the smelting process.

### DT application system

Currently, DT technology is widely used in aerospace, bridge construction, transportation, healthcare, intelligent manufacturing, human-machine collaboration, metal smelting, physical networks, energy, power, as well as training and education industries.

Yang et al. [65] classified the service types of DT systems into three categories: equipment/component, production line/process, and factory/city. The architecture of DT system can also be classified into unit-level, system-level, and system-of-systems level. A unit-level DT system is built based on manufacturing units, including virtual manufacturing objects and resources. A system-level DT system is built by combining multiple manufacturing units through a communication network. A system-of-systems level is built by connecting system-level and unit-level DT manufacturing systems through an intelligent platform.

Considering the focus of application requirements, a DT is classified according to the scope covered by the DT object into three categories: unit-level, process-level, and system-level DTs (Table 2).

**Table 2:** Status of research on DT application systems

Hierarchy of application system	Application areas of the system	Reference	Function of the application system
Unit-level DT	Aerospace	[43, 66–68]	Life prediction Damage prediction
	Bridge construction	[69, 70]	Building structural health monitoring Life prediction
	Transportation	[71–73]	Virtual testing Vehicle health monitoring
	Healthcare	[74, 75]	Surgical or medical simulation
Process-level DT	Intelligent manufacturing	[12, 76–82]	Production process simulation Diagnosis of machine faults Monitoring and forecasting of production tools or machines
	Human-machine collaboration	[55, 83–87]	Optimization of industrial human–machine collaboration
	Metal smelting	[88, 89]	Production process simulation
	Physical networks	[90–92]	Implementation of Industry 4.0, IoT and cyber-physical production systems
System-level DT	Smart factory	[93–97]	Improving the efficiency of shop floor manufacturing equipment Optimizing production planning and resource allocation Operational monitoring and troubleshooting
	Training and education	[98–101]	Optimizing distance learning or online teaching
	Energy and power	[58, 102–106]	Optimizing production planning and resource allocation Operational monitoring and troubleshooting
	Intelligent transportation	[107]	Increasing traffic condition awareness to aid driving decisions

#### Unit-level DT

The unit-level DT is designed for individual parts and products. Users create their DT to perform virtual testing and performance prediction to improve the safety and reliability of the equipment. Unit-level DTs are typically used in the following applications:

##### Aerospace

Tuegel et al. [43] retested aircraft structural life prediction to take advantage of high performance numerical computation. A conceptual DT model for aircraft structural life prediction and structural integrity verification was proposed. Li et al. [66] predicted and visualized fatigue crack extension by establishing a diagnostic and prognostic probabilistic model for aircraft wings. Bayer et al. [67] developed a metamodeling approach for dynamic systems, producing models that could be used for fast stochastic analyses and dynamic real-time experiments. Millwater et al. [68] performed a fracture probability treatment of critical component locations for the reliability, safety, and economy of the fuselage DT and its practical equivalents.

##### Bridge construction

Omer et al. [69] proposed a bridge-inspection method. The bridges were digitized using LiDAR. A case study involving a typical masonry bridge was evaluated using VR. Shim et al. [70] proposed a new generation of bridge preventive maintenance systems. A DT model was used for more reliable decision making. A 3D information model-based maintenance information management system was combined with a digital inspection system using image processing, by continuously exchanging and updating the data from each stakeholder.

##### Transportation

Venkatesan et al. [71] created an intelligent DT using the MATLAB/Simulink software, which was previously used for health monitoring and prognostic analysis of electric vehicle motors. Shcherba et al. [72] applied a DT to vehicle crash detection. The damage-theory-based GISSMO failure description method was applied to the entire vehicle model, obtaining a good correlation with full-scale crash tests at high strain rates. Korostelkin et al. [73] developed a DT body to reduce the cost of off-road vehicle body-quality inspection. Local and overall body stiffness, strength constraints, and crash safety requirements were considered.

##### Healthcare

DT and healthcare have been combined to provide a new and efficient healthcare delivery method. However, achieving personal health management for the entire life cycle of patients and integrating the physical world of healthcare with the virtual world to achieve truly intelligent healthcare remain the two key challenges in the era of precision medicine. Liu et al. [74] proposed a novel, generic, and scalable framework for a DT-based cloud health system to monitor, diagnose, and predict all aspects of an individual’s health. Pizzolato et al. [75] discussed the integration of real-time neuromusculoskeletal system models with finite elements of musculoskeletal tissues. In this study, a model of the neuromusculoskeletal system was developed to optimize muscle stimulation patterns, track functional improvements, monitor safety, and provide enhanced feedback during exercise-based rehabilitation.

#### Process-level DT

Process-level DT systems involve the development of processes consisting of multiple parts or products. Users create DTs and use DT models for simulation and control to improve performance, such as controllability and visualization. Process-level DTs are typically used in the following applications:

##### Intelligent manufacturing

The manufacturing industry has moved away from purely physical-mechanical processing and entered an era of interaction and iteration between physical and digital worlds [76, 77]. Therefore, integration of the physical and digital manufacturing spaces is required. The development of DT technology facilitates the realization of this goal [12, 78]. Liu et al. [79] used a DT for marine diesel engine production process evaluation to improve product quality and shorten the development cycle. Three core technologies were developed: a real-time mapping mechanism between data collected in machining and process design information, construction of a DT-based machining process evaluation framework, and process evaluation driven by DT data. Rauch and Pietrzyk [80] introduced DTs into the manufacturing process of high-strength steel strips. A virtual rolling line consisting of basic equipment, such as a heating furnace, descaling machine, rolling stand, laminar flow cooling, and coiler was designed. Yerra and Pilla [81] proposed a new assembly line layout using virtual factory simulation tools to break down the traditional automotive manufacturing process and layout. Based on a DT, Dong et al. [82] proposed a relational functional model for a hierarchical functional backtracking product redesign method.

##### Human-machine collaboration

Oyekan et al. [83] investigated the effectiveness of using virtual environments to develop human-robot collaboration strategies in response to the unpredictability of accidents during human-robot collaboration. Malik and Bilberg [55] proposed a DT framework to support the design, construction, and control of human-robot collaboration. Bilberg and Malik [84] designed a DT of a flexible assembly cell to enable robots to collaborate in assembly tasks. Hu [85] developed a DT with real-time interactive information gain and visualization templates through bidirectional data flow and real-time optimization to reduce the uncertainty of the sensory-motor processes. Lee et al. [86] developed and tested a DT deep reinforcement learning (DRL) method to explore the potential of DRL for adaptive task assignments in robotic construction environments. Li et al. [87] proposed a DT-based safety control framework and corresponding control methods to test and analyze potential safety hazards.

##### Metal smelting

Gupta and Basu [88] used a DT to continuously generate new data to gain insight into aluminum smelter performance, predict potential challenges, suggest operational remedies, and generate process controls. Llamas et al. [89] used simulation models for Zn production to evaluate material recovery, resource consumption, and environmental impacts of different processing routes.

##### Physical networks

Dai and Burns [90] proposed an online adaptive approach based on a DT for long-lived, uninterrupted cyber-physical system reliability problems. A DT model and historical data were used to achieve real-time tuning. Arafsha et al. [91] created a DT of physical devices, mirroring their attributes and sensory information in the cyber world for real-time analysis. Dong et al. [92] used the DT of a real web environment for offline training on a central server to optimize user association and complete resource allocation.

#### System-level DT

System-level DTs cover the complete process of existence and development of entities. Users create their DT and use it for simulation, prediction, and scheduling to improve the controllability and visualization of the twin system. Process-level DTs are typically used in the following applications:

##### Smart factories

Söderberg et al. [93] investigated the application of DTs for product development and production engineering. The control and optimization of the production process were achieved using a real-time simulation. Sierla et al. [94] used a DT to develop assembly plans and coordinate production resources. Fang et al. [95] proposed a new method for job shop scheduling based on a DT to achieve real-time and accurate scheduling. Longo et al. [96] used DT technology to control the production cost and process quality of manufacturing systems. Zhang et al. [97] proposed a dynamic resource-allocation model for a DT-driven smart shop to achieve real-time data collection and dynamic simulation.

##### Training and education

DT technology provides users with new experiences that are impossible to realize in the real world. Nikolaev et al. [98] created a DT for real products. Innovative product design courses based on real-world case studies have also been developed. Kim et al. [99] used DT technology to integrate the real world into VR, and realized efficient teaching and learning based on VR on a mobile platform. Toivonen et al. [100] created a generic learning environment for flexible manufacturing systems that allowed students to familiarize themselves with fully automated production systems, developing and testing programs in a virtual environment. Verner et al. [101] proposed a connected environment that integrates robots, DTs, and virtual sensors.

##### Energy power

Tao et al. [58] used DT technology to predict and manage the health of a wind turbine. Biglarbegian [102] completed reliability sensing of GaN devices in high-frequency power converters using a DT. Zhou et al. [103] completed the online analysis of a power grid for the development of a new real-time online power grid analysis system. Zhou et al. [104] established a real-time online analysis platform based on DT technology to shorten the online analysis cycle of power grids. He et al. [105] verified the reliability of DT-based power system trend analysis. Francisco et al. [106] developed daily building energy benchmarks based on strategic periods, using smart meter power data to quantify the differences from traditional annual energy benchmarking strategies.

##### Intelligent transportation

Kumar et al. [107] built an intelligent infrastructure system which fills the gap in vehicle perception and extends the horizon by creating a form of a DT model for current traffic conditions. Ground-truth data were generated using aerial imagery and Earth observation methods to evaluate the spatial accuracy and recall of the DT model.

## Conclusions

A DT serves as a digital representation of units, processes, and systems. This enables the linking of various stages, thereby increasing efficiency, reducing failure rates, and shortening development cycles. DT provides a new and effective means of observing, recognizing, understanding, controlling, and transforming the physical world.

Despite the strategic importance of the DT, designing DT systems remains a complex process for organizations in any industry. There is a huge gap between the promising prospects depicted by the DT and realistic technology level in industry and equipment. In this study, DT technology related application research was analyzed and it was concluded that there are three main challenges:

1. The virtual modeling technology of DTs must be enhanced. DT engineering remains a complex process in any type of industry. It involves not only the spatial modeling of the geometric structure and positional relationships of objects but also the logical modeling of mechanistic and regular models of these entities. This development process requires interdisciplinary cooperation among the various engineering fields. However, there are differences in scholars’ understanding of the same entity in different fields, and the degree of collaboration among experts in multiple fields has a significant impact on the consistency of the DT with physical entities. In addition, dedicated tools and platforms exist for each field, such as Simulink in MATLAB, Twin Builder in ANSYS, Azure in Microsoft, and 3D Experience in Dassault. However, the combination of tools and methods from different domains is inadequate. Presently, holistic and convergent virtual modeling techniques and tools that can be useful throughout the digital life cycle are lacking.
2. The evaluation criteria for DTs should be more rigorous. Depending on the creation method, multiple types of DTs can be generated for the same object. Some scholars have actively explored the evaluation criteria of DTs [50, 51]. However, systems that evaluate the degree of development of existing DTs and clarify the direction of DT construction to guide upgrading and optimization, are still lacking.
3. The theoretical foundation of DTs must be strengthened. Although DTs have received considerable attention in recent years, this is an emerging research direction, and the concept is developed only after practice. However, the emerging research direction of “practice first, concept later,” which is often attached to information technology, big data, artificial intelligence, and IoT in the development process, lack a relevant theoretical basis in research. The research results are directly applied to various engineering practices [108]. Although this approach helps promote DT technology, it lacks theoretical foundation.

DT technology has good prospects in intelligent manufacturing and equipment maintenance. It is gradually receiving attention from both military and civilian sectors, including robotics, aerospace, new energy, and other industries. All of these sectors have started to explore technical systems, key technologies, and application potentials of DTs.

The future development trend of DT is predicted to follow two directions:

1. *Integration of related technologies*. The implementation of DT technology relies on industrial information systems, artificial intelligence, big data, and other technologies. However, despite the rapid development of these technologies, DT technology is still active and emerging. Better utilization of research results of other related technologies in DTs will be one of the main research directions in the future.
2. *Continuous improvements in industrial applications*. With the development of industrial technology and requirements, equipment design, testing, operation, maintenance, and other life cycle costs have increased significantly. Concurrently, the complexity of equipment has considerably increased the chances of performance degradation and functional failure. Inspired by practical considerations, DTs of complex equipment will become the focus of future research. Various engineering practitioners are exploring and experimenting with optimization and refinements so that the scope of DT applications can be expanded.

This paper summarizes the development history, definition, and application areas of DTs, thereby proposing the definition of a DT and DT model based on TSL. It is expected that the analysis and summary of this study will provide further ideas and references for the development and application of DT technology.

## Data Availability

Not applicable.

## Abbreviations

AR: Augmented reality
CAD: Computer-aided design
CAE: Computer-aided engineering
DT: Digital twin
DRL: Deep reinforcement learning
ECDTJ-DC model: Edge-computing-based dynamic scheduling model
IoT: The internet of things
TSL model: Time, space, and logic model
VR: Virtual reality
XR: Extended reality
